# Evolutionary history of the fish genus *Astyanax *Baird & Girard (1854) (Actinopterygii, Characidae) in Mesoamerica reveals multiple morphological homoplasies

**DOI:** 10.1186/1471-2148-8-340

**Published:** 2008-12-22

**Authors:** Claudia Patricia Ornelas-García, Omar Domínguez-Domínguez, Ignacio Doadrio

**Affiliations:** 1 Departamento de Biodiversidad y Biología Evolutiva, Museo Nacional de Ciencias Naturales, CSIC, José Gutiérrez Abascal 2, Madrid, Spain; 2 Posgrado en Ciencias del Mar y Limnología, Instituto de Ciencias del Mar y Limnología, Circuito exterior s/n, Ciudad Universitaria, México DF, Mexico

## Abstract

**Background:**

Mesoamerica is one of the world's most complex biogeographical regions, mostly due to its complex geological history. This complexity has led to interesting biogeographical processes that have resulted in the current diversity and distribution of fauna in the region. The fish genus *Astyanax *represents a useful model to assess biogeographical hypotheses due to it being one of the most diverse and widely distributed freshwater fish species in the New World. We used mitochondrial and nuclear DNA to evaluate phylogenetic relationships within the genus in Mesoamerica, and to develop historical biogeographical hypotheses to explain its current distribution.

**Results:**

Analysis of the entire mitochondrial cytochrome *b *(*Cytb*) gene in 208 individuals from 147 localities and of a subset of individuals for three mitochondrial genes (*Cytb*, 16 S, and *COI*) and a single nuclear gene (*RAG1*) yielded similar topologies, recovering six major groups with significant phylogeographic structure. Populations from North America and Upper Central America formed a monophyletic group, while Middle Central America showed evidence of rapid radiation with incompletely resolved relationships. Lower Central America lineages showed a fragmented structure, with geographically restricted taxa showing high levels of molecular divergence. All *Bramocharax *samples grouped with their sympatric *Astyanax *lineages (in some cases even with allopatric *Astyanax *populations), with less than 1% divergence between them. These results suggest a homoplasic nature to the trophic specializations associated with *Bramocharax *ecomorphs, which seem to have arisen independently in different *Astyanax *lineages. We observed higher taxonomic diversity compared to previous phylogenetic studies of the *Astyanax *genus. Colonization of Mesoamerica by *Astyanax *before the final closure of the Isthmus of Panama (3.3 Mya) explains the deep level of divergence detected in Lower Central America. The colonization of Upper Mesoamerica apparently occurred by two independent routes, with lineage turnover over a large part of the region.

**Conclusion:**

Our results support multiple, independent origins of morphological traits in *Astyanax*, whereby the morphotype associated with *Bramocharax *represents a recurrent trophic adaptation. Molecular clock estimates indicate that *Astyanax *was present in Mesoamerica during the Miocene (~8 Mya), which implies the existence of an incipient land-bridge connecting South America and Central America before the final closure of the Isthmus of Panama (~3.3 Mya).

## Background

Mesoamerica is one of the most complex biogeographical areas in the world [1-5]. This complexity reflects the confluence of Neotropical and Nearctic biotas and a long history of geological activity, stretching from the Miocene to the present, during which movements of the Cocos, North American, Pacific and Caribbean Plates [6,7] created barriers and land-bridges that have affected the distribution of freshwater fishes [8-13]. For example, the Pliocene (~3.3 Mya) closure of the Panama Strait has been postulated to be one of the most important causes of faunal interchange between Neartic and Neotropical regions [14]. Climatic changes have also been invoked to explain the distribution of Mesoamerican fish fauna [15]. Distinguishing between climatic and geological effects requires information on phylogeny and species boundaries in a diversity of taxa [8,9,16-19].

Special attention has been devoted to understanding the number and timing of colonizations of Mesoamerica by freshwater fishes from South America: a topic which remains somewhat controversial [8,13,20-22]. The most widely accepted theories support two waves of colonization: 1) an ancient episode (70 – 80 Mya) through a proto-Antillean arc and 2) a more recent, Cenozoic episode via the Antillean islands and/or a continental corridor [14,23,24]. Molecular data suggest colonization of Mesoamerica by primary freshwater fishes about 4–7 Mya [8,12,13,22]. This is incongruent with the geological data, which does not support the existence of a continental land-bridge before the closure of the Panama Strait (3.3 Mya). Older colonization events have been postulated for secondary freshwater fishes: for example, Early to Mid Miocene (12.7–23 Mya) colonization for Synbranchidae [13], 14–24 Mya for heroinid cichlids (10 Mya for Mesoamerican lineages) and 18.4–20 Mya for rivulids [22].

The absence of primary freshwater genera (e.g., *Hypopomus*, *Pimelodella*, *Rhamdia *and *Roeboides*) from Mesoamerica and the Antillean islands argues against an ancient colonization route through a proto-Antillean arc. Instead, it supports a colonization route through an incipient land-bridge formed during the gradual uplifting of the Panama Isthmus [8,12], over a time span of 3–20 Mya, combined with changes in sea level [25]. This is supported by molecular studies of arthropods [26], amphibians [27], and marine geminate species pairs on either side of the Panamanian Isthmus [28].

Support for a more recent colonization of Mesoamerica by primary freshwater fish through the Panama Strait comes from phylogeographical studies of Characids (e.g., *Brycon, Bryconamericus, Eretmobrycon*, and *Cyphocharax*). These studies indicate multiple waves of rapid expansion from South America during the Pliocene ~3.3 Mya. [15].

The genus *Astyanax *provides an ideal model to investigate the relative importance of vicariance and dispersal on biogeographical patterns. This is partly because it is widely distributed across the region [29], and because its dispersal is confined to freshwater routes and dependent, therefore, on the formation of land-bridges.

Characiforms are generally assumed to have a Gondwanan (South American) origin [30-32], as supported by the fossil record [33], so the presence of Characidae in Northern America is viewed as a consequence of dispersal.

*Astyanax *comprises more than 107 recognized species and is, together with *Hyphesobrycon *(105 species), the largest and most diverse characiform genus [34,35]. Moreover, *Astyanax *has the widest distribution of American characids, being found from the Nearctic (Colorado River in Texas and New Mexico) to the Neotropics (Negro River in Patagonia) [4].

Previous phylogenetic studies [36,37] of the biogeography of *Astyanax *used a small number of samples from Mexico, Belize and Guatemala, and did not find geographical congruence for some of the groups recovered (i.e. Yucatan and Belizean populations were not the most closely related despite their geographical proximity). Furthermore, conspecific cave and nearest surface populations formed two separate lineages, in agreement with an earlier study of the genus [38]. This was attributed to at least two separate colonizations of Mesoamerica from South America during the Pleistocene. Estimated colonization times based on the cytochrome *b *gene were 1.8 and 4.5 Mya (3.1 Mya), with an estimated divergence rate of 1.5% per pairwise comparison per million years, which coincides with the closure of the Panama Strait (3.3 Mya) [37]. However, incomplete sampling (only few samples were included from upper Central America and Mexico) could lead to erroneous interpretations. This study provides a phylogeographical analysis based on a comprehensive distribution-wide sampling regime and more extensive sampling, and thus should provide new insights into the evolutionary history of the genus.

The genus *Astyanax *is characterised by high phenotypic plasticity and a capacity to adapt to diverse habitats [36,38-41]. There is clear evidence of extremely rapid adaptations of fish to new habitats and environments, with ecological specialization and morphological differentiation, generally in accordance with genetic divergence [15,42-44]. Considerable attention has been given to the evolution of developmental mechanisms and adaptation to cave environments [36,38,39], but less attention has been given to other habitat associated morphological plasticity. In this regard, *Bramocharax*, which is sympatric with *Astyanax*, is characterized by conspicuous trophic specializations, including differences in the number of premaxillary teeth, the presence of diastemas on the maxillary teeth, as well as differences in the shape and number of cuspids on the premaxillary, maxillary and dentary teeth, with some species (*B. caballeroi *and *B.baileyi*) having intermediate states between the morphotypes of *Astyanax *and *Bramocharax *species [45-47].

In this study we used mitochondrial and nuclear DNA sequences to develop a robust phylogenetic hypothesis for *Astyanax *and *Bramocharax*. This allows us to test biogeographical hypotheses for the Mesoamerican fish fauna, including the relative importance of historical geology and climatic factors.

## Results

Three mitochondrial (*Cytb, COI *and 16 S) genes and one nuclear gene (*RAG-1*) were sequenced, giving a total of 3862 characters (2350 mitochondrial and 1512 nuclear).

*RAG-1 *was the most conservative of the genes analyzed (Table 1). Among the mitochondrial DNA genes, *Cytb *was the most variable, with *COI *exhibiting similarly high levels of variability and 16 s being the least variable. For the joint mitochondrial and nuclear analysis, 931 sites were variable, with 448 (~11%) being parsimony informative.

**Table 1:** Primers and PCR conditions.

Gene	Primers	Sequence (5'-3')	Tm (°C)	Size (pb)	Reference
*RAG1*	RAG1 (a, s)	AGCTGTAGTCAGTAYCACAARATG			
	RAG5 (s)	TRGAGTCACACAGACTGCAGA	58*	1512	[67].
	RAG9 (a, s)	GTGTAGAGCCAGTGRTGYTT			
Cytochrome oxidase I (*COI*)	FISHF1 (a, s)	TCAACCAACCACAAAGACATTGGCAC			
	FISHR1 (a)	TAGACTTCTGGGTGGCCAAAGAATCA	54	655	[83]
Cytochrome *b *(*Cytb*)	Glu- F (a, s)	GAAGAACCACCGTTGTTATTCAA			
	Thr- R (a, s)	ACCTCCRATCTYCGGATTACA	48		[84]
	CbCHR (a, s)	TTARTCCGGCTGGGWTNTTTG	48	1140	This study
16 S	16SAR (a, s)	CGCCTGTTTATCAAAAACAT			
	16SBR (a)	CCGGTCTGAACTCAGATCACGT	46	552	[85].

Amplification (a) and sequencing (s) primers used for the *RAG1*, 16S, *COI*, and *Cytb *genes. *Touchdown PCR was performed for the first 10 cycles: from 58°C to 53°C (-0.5°C each cycle), followed by 25 cycles at 53°C.

**Figure 1:**
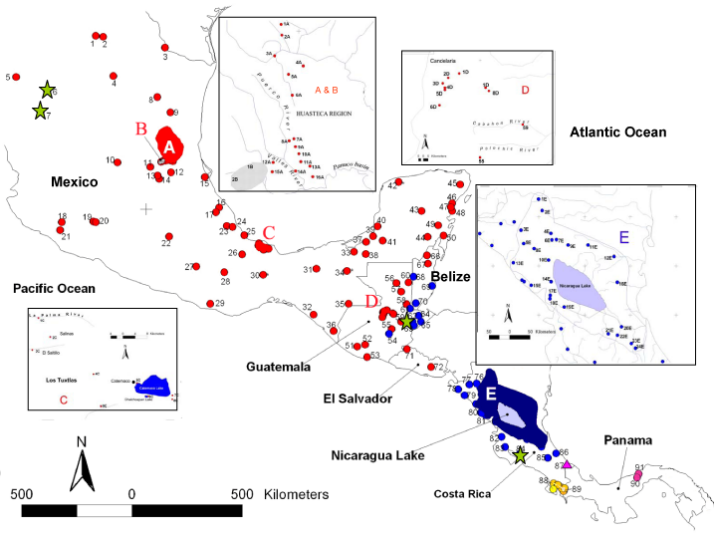
**Sampling sites**. Map of the sampled localities in Mesoamerica. The six major groups obtained in our phylogenetic analyses are represented by different colours. Stars represent the localities or basins where we found different lineages in sympatry.

The topologies recovered by Maximum Parsimony (MP) and Bayesian Inference (BI) for the *Cytb *data set (Figure 1) and combined data set were similar. In addition, *Cytb *and the combined data set analyses were also concordant, with discrepancies restricted to the tree topologies in Clades III and IV from Group I (the Maquinas population was grouped with Montebello in the *Cytb *topology but with Polochic-Grijalva-Usumacinta with the combined data set). The combined data matrix was useful to resolve the deeper nodes and recovered mostly higher support values, providing greater phylogenetic resolution. For this reason, description of the higher-level groups identified was based on the topology obtained with the combined data set.

All analyses supported the polyphyly of *Bramocharax*, with species of *Bramocharax *being sister groups to different clades of *Astyanax *(Figure 1), making *Astyanax *paraphyletic.

**Figure 2:**
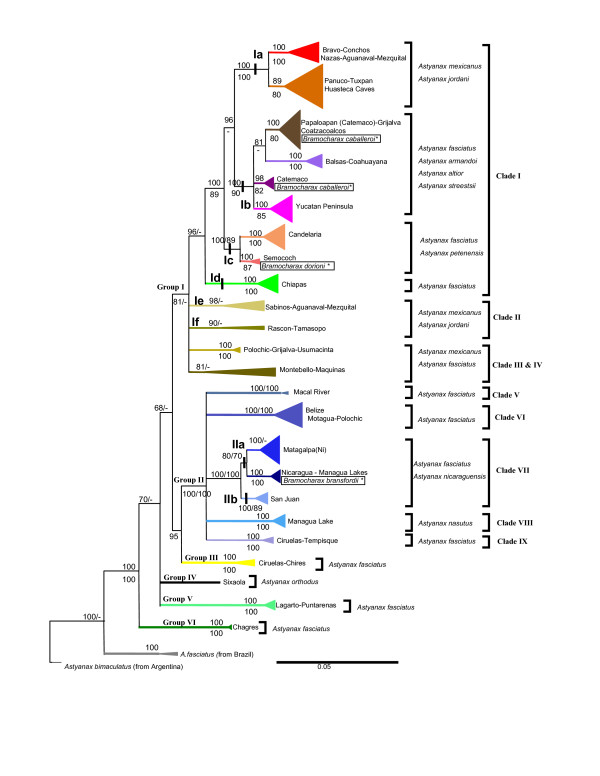
**Summarized Phylogenetic tree estimated with Bayesian Inference and maximum-parsimony methods using the *Cytb *gene**. Bayesian and Maximum-Parsimony inference tree for *Astyanax *and *Bramocharax *genera (the latter denoted by squares) based on the *Cytb *gene. The posterior probabilities and bootstrap values are shown. The species considered as valid according to Lima *et al*. [35] for each linage are also shown. Definition of the Clades was based on the Combined data set tree.

We identified six major phylogenetic groupings with high bootstrap support and significant posterior probabilities (Figures 1 and 2). Percentage divergences between groupings are given in Table 2. Groups V and VI correspond to the Chagres region (Panama) and Lagarto-Puntarenas basins of Costa Rica in Lower Central America, respectively. Groups II to IV are from Middle Central America and Group I is from Upper Central America and Mexico. These groups are non-overlapping geographically except for I with II and II with III (see phylogenetic Clades scheme in Additional file 1). Their inter-relationships were not resolved with either the *Cytb *or the combined data set (3.8 Kbp).

**Table 2:** Phylogenetic Performance of each gene.

Gene	Size (pb)	Variable sites	PI	% PI	CI	RI
*Cytb*	1140	505	298	26.14	0.58	0.85
*COI*	655	137	93	14.19	0.46	0.64
16S	555	74	19	3.42	0.52	0.61
*RAG1*	1512	215	38	2.51	0.56	0.07
All	3862	931	448	11.09	0.73	0.61

Molecular characterization for the data set for each gene. PI = parsimony informative characters, CI = Consistency index, RI = retention index.

### Geographic structuring within the major phylogenetic groupings

#### GROUP I (Mexico and Upper Central America)

Four main clades were recovered from Group I. Clade I comprised most of the Mexican and Upper Central American (Guatemala and Belize) populations. Clades II-IV represented fewer populations with a patchy distribution over the range of Clade I.

Within Clade I we found geographical structure corresponding to the following four lineages: Lineage Id, from the Chiapas region of Mexico, was sister to a clade comprising Lineage Ie from the Candelaria region and a pair of sister lineages (1a and 1b) that occupy a wider region from the Yucatan Peninsula to the Bravo-Conchos basin.

**Figure 3:**
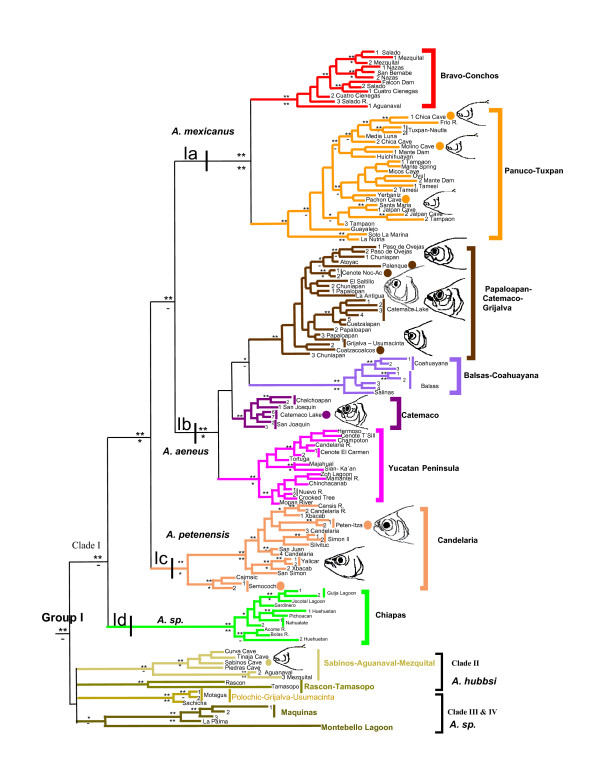
**Subtree of Group I based on *Cytb *gene**. Subtree of Group I for the Bayesian Inference and Maximum-Parsimony methods for *Astyanax *and *Bramocharax *based on the *Cytb *gene. Double asterisks indicate Bayesian posterior probabilities ≥ 0.95 or MP bootstrap values ≥ 90; single asterisks identify values between 0.89 and 0.80 or 89 and 80. Circles represent type localities. Definition of the Clades was based on the Combined data set tree.

Lineage Ia contained individuals of *A. mexicanus *and the troglobitic species *A. jordani *from northern Mexico. This lineage was subdivided in two sublineages. The first included samples from Bravo-Conchos basin in the northern-most part of the range of *Astyanax*, and the basins of Mezquital and Nazas – Aguanaval. The second sublineage grouped populations from the Panuco, Tuxpan, Nautla and San Fernando-Soto La Marina basins, including most troglobitic populations from the Huasteca region (see region A in the Figure 3). Therefore, this latter sublineage included *A. mexicanus *and the cave-dwelling nominal species, *A. jordani*.

Lineage Ib contains *Astyanax *and *Bramocharax *from southern TMVB and Belize. It is subdivided into four sublineages. It ranges from the Media Luna Lagoon (Panuco basin, Mexico) to the Mopan Basin (Belize) on the Atlantic slope, and from the Armeria – Coahuayana Basin to the Balsas Basin (both in Mexico) on the Pacific slope. This group is a good example of morphological plasticity with low levels of genetic differentiation (see further details in Additional file 2).

**Figure 4:**
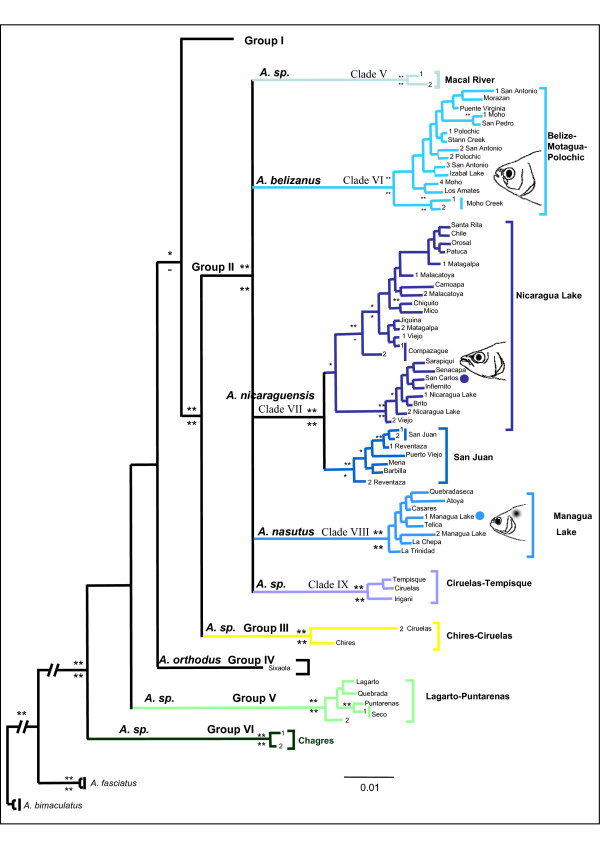
**Subtree of Groups II to VI based on *Cytb *gene**. Subtree of Group II – VI for the Bayesian Inference and maximum-parsimony methods for *Astyanax *and *Bramocharax *based on the *Cytb *gene. Double asterisks indicate Bayesian posterior probabilities ≥ 0.95 or MP bootstrap values ≥ 90; single asterisks identify values between 0.89 and 0.80 or 89 and 80. Circles represent type localities. Definition of the Clades was based on the Combined data set tree.

The first sublineage included *Astyanax fasciatus *from Puente Nacional to Grijalva – Usumacinta basins (including Papaloapan and Coatzacoalcos basins) on the Atlantic slope, and populations from the type localities of *A. armandoi *and *A. altior *(Palenque in the Grijalva – Usumacinta basin and Cenote of Noc-Ac in the Yucatan Peninsula, respectively). Both of these are junior synonyms of *A. fasciatus *[35,48]. We also found shared haplotypes or low divergence (see Additional file 2) between *Bramocharax caballeroi *and sympatric *A. fasciatus *from Lake Catemaco (see region C, Los Tuxtlas, Figure 4). Additionally these distances were lower than those observed within *Astyanax *populations.

The second sublineage (Catemaco) was a shallow clade restricted to the Tuxtlas region (lakes Chalchoapan and Catemaco, Figure 3), and comprised morphotypes of *A. fasciatus *and *B. caballeroi*, with very low genetic distinctiveness with haplotypes shared in some cases (Figures 1 and 2, see also Additional file 2). The third sublineage included *A. fasciatus*, mostly from the Pacific slope (Coahuayana to Balsas basins), and one single population of *A. mexicanus *from the Media Luna Lagoon basin on the Atlantic slope (Figure 3). The fourth sublineage ('Yucatan Peninsula', Figure 1) included *A. fasciatus *from Grijalva-Usumacinta (Candelaria River), Cenotes from Yucatan Peninsula, Belize and Nuevo basins (Belize) and Mopan basin (Guatemala).

Lineage Ic, (region D, Figure 3) comprised populations from the Atlantic slope: *Astyanax fasciatus *(a synonym of *A. mexicanus *sensu Lima *et al*. [35]) from the type locality of *A. petenensis *(Peten – Itza Lake, Yucatan Peninsula) and *A. fasciatus *from the Candelaria karst region in Guatemala. This lineage included *Bramocharax dorioni *from its type locality (Semococh river). Similar to other instances of sympatry between *Bramocharax *and *Astyanax*, these two morphotypes showed low levels of genetic differentiation (less 0.5%, see Additional file 3).

Lineage Id (Chiapas) grouped Pacific slope populations of *A. fasciatus *(Figure 1) from the Pichoacan basin (Oaxaca, Mexico) to the El Jococal Lagoon (El Salvador), including the Guatemalan coast, with very low genetic divergence within the clade.

Clade II comprised two lineages. (Ie and If; mean divergence of ${\bar{\text{D}}}_{K81uf}$ = 2.13% ± 1.21). Lineage Ie (referred to as "Sabinos-Aguanaval-Mezquital") grouped troglobitic morphotypes (*A. jordani*) from the Piedras, Tinaja, La Curva, and Sabinos caves (type locality of *A. hubbsi*, synonym of *A. jordani sensu *Lima *et al*. [35]) with the surface-dwelling populations of *A. mexicanus *from the Mezquital and Nazas – Aguanaval basins, with a low level of differentiation between surface and cave populations (mean of ${\bar{\text{D}}}_{K81uf}$ = 1.4% ± 0.9).

Lineage If included populations of *A. mexicanus *from the Rascon valley and the Panuco basin in Tamposa. This lineage was highly divergent with regard to the rest of the lineages in this group, and those populations closest geographically (see Additional file 2)

Clade III grouped Atlantic slope populations of *A. fasciatus *from the La Palma and Maquinas basins (see Los Tuxtlas region, Figure 3) with those from the upper Polochic basin (Cahabon river) and the Grijalva-Usumacinta basin.

Clade IV contained populations from the Montebello Lagoons (Montebello) in south-eastern Mexico. This group clustered with the Maquinas and La Palma populations in the analysis of *Cytb *alone, but this clustering was not supported by the combined nuclear and mitochondrial analysis (Figures 2 and 4).

#### GROUP II (Middle Central America)

Our analyses did not resolve relationships among the five main clades recovered within Group II. This group occurs widely over Middle Central America, ranging from Belize to Nicaragua and Costa Rica (E, Figure 3).

Clade V included *A. fasciatus *from the Macal Basin (Belize). It is highly divergent with respect to the other clades from Group II (D_*K81uf *_= 3.6% ± 1.39).

**Figure 5:**
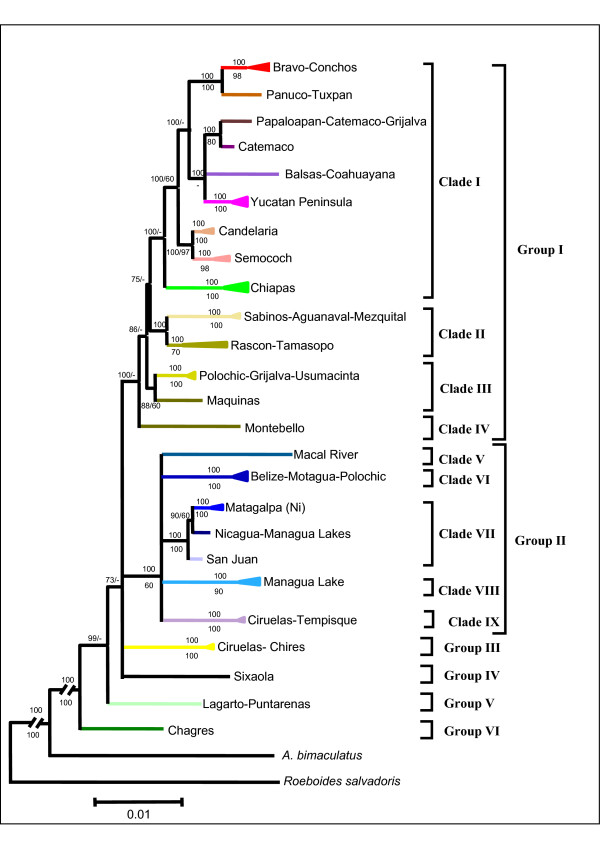
**Combined data set tree**. Summarized Phylogenetic tree for *Astyanax *and *Bramocharax *estimated with Bayesian Inference and Maximum-Parsimony methods using the subset of data (*Cytb*+*COI*+16S and *RAG1*). Posterior probabilities and bootstrap values are shown.

Clade VI ("Belize-Polochic-Motagua") includes Atlantic slope *A. fasciatus *from the Moho basin (Belize), and from Guatemala, downstream of the Polochic Basin to the Puente Virginia Basin (Figure 5).

Clade VII comprised two lineages (IIa and IIb). Lineage IIa included *Astyanax nicaraguensis *and *Bramocharax bransfordii*. As in other clades containing both morphotypes there were low levels of genetic differentiation (D_*K81uf *_= 0.9% ± 0.61). This lineage occurs from the Jigüina Basin to the Sarapiqui basin (Nicaragua) on the Atlantic slope, and included populations from the great Lakes of Nicaragua (Managua and Nicaragua, region E, figures 1 and 4) and the Senacapa and Brito basins (Nicaragua) on the Pacific slope. Lineage IIb included *A. fasciatus *from Lake Nicaragua and the San Juan basin (Nicaragua), as well as the Barbilla and Reventaza basins on the Atlantic slope of Costa Rica.

Clade VIII included *A. fasciatus *from the Pacific tributaries of Nicaragua and *A. nasutus *from the Managua Lake Basin.

Clade IX included *A. fasciatus *from a very restricted area comprising the Ciruelas and Tempisque basins on the Pacific slope of Costa Rica ("Ciruelas-Tempisque", Figure 5).

### Lower Central America

Four major groups (groups III-VI) in Lower Central America were characterized by more restricted ranges relative to Groups I and II. Group III comprised *A. fasciatus *populations from the Ciruelas and Chires basins on the Pacific Slope of Costa Rica (figures 1 and 2). Two highly divergent (D_*K81uf *_= 2.5%) haplotypes (corresponding to Groups II and III) were found in sympatry in the Ciruelas basin (figures 1 and 2).

Group IV included a single and well-differentiated population of *A. orthodus *from the Sixaola basin on the Atlantic slope of Costa Rica. Group V contained Pacific slope *A. fasciatus *populations from the Puntarenas basin (Costa Rica) to the Lagarto basin on the Panama-Costa Rica border. Group VI included *A. fasciatus *from the Chagres region on the Atlantic slope of Panama.

## Discussion and conclusion

### Systematics of the genera *Astyanax *and *Bramocharax*

Our analyses do not support the previously proposed monophyly of *Bramocharax *based on morphological analyses [45-47]. Moreover, *Bramocharax *specimens were present in two of the seven major *Astyanax *clades, with low levels of genetic differentiation when both morphotypes were found in sympatry (such as in lake Catemaco where it was possible to find haplotypes shared between individuals from both genera). Differentiation was equally reduced between allopatric populations of *Bramocharax *and *Astyanax*.

The genus *Astyanax *has been considered to be monophyletic in Mesoamerica [37], but polyphyletic in South America [30] on the basis of molecular analyses. Our results support the monophyly of Mesoamerican *Astyanax *only if we consider *Bramocharax *species to be morphotypes of *Astyanax *within the range of its phenotypic plasticity. This hypothesis is supported by the low genetic divergence between specimens of *Bramocharax *and *Astyanax*, and the evidence of recurrent evolution of the *Bramocharax *morphotype within *Astyanax *(Figure 3). This morphotype is associated with lacustrine habitats, suggesting that its recurrent evolution is a result of morphological convergence to similar ecological factors; similar patterns have been shown in other freshwater fishes [37,38,41,45,49]. If the "recurrent convergence" hypothesis is considered to be correct, then the taxonomy of *Bramocharax *needs to be revised and the evolutionary mechanisms giving rise to these morphological homoplasies need further investigation. Our analyses question the taxonomic utility of trophic characters (e.g., teeth shape or jaw modification), as previously done by Rosen [47] on the basis of intermediate morphological states between *Astyanax fasciatus *and *Bramocharax baileyi*.

**Figure 6:**
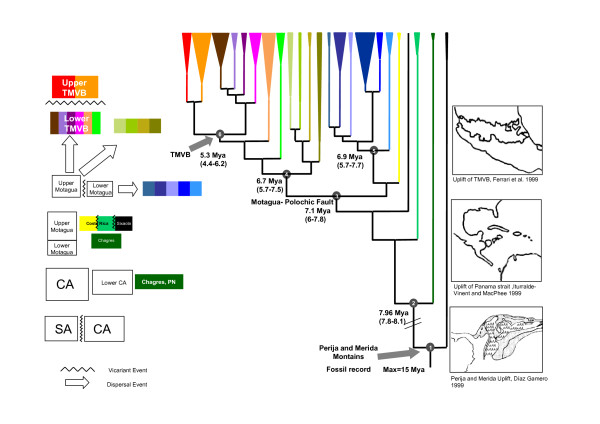
**Ultrametric tree based on *Cytb *topology using semi-parametric penalized likelihood**. Ultrametric tree based on the topology obtained with the mitochondrial *Cytb *gene using semi-parametric penalized likelihood. The calibration points are indicated by arrows, the first, node 1, corresponds with the rising of the Sierra of Perija and Merida Mountains Díaz de Gamero [79], and the second, node 6, corresponds to the final closure of the Trans-Mexican Volcanic Belt Ferrari *et al*. [82]. The main vicariant events are shown in the diagram as are the dispersal events.

Further incidences of morphological convergence were found in troglobitic morphotypes of *Astyanax jordani *(this has been noted by previous authors [37,38,50]), providing further evidence of independent (at least two different times, see Figure 6) adaptation to troglobitic habitats. The presence of recurrent morphological convergence in *Astyanax *[50,51] makes the delimitation of species and genera difficult. Thus the absence of congruence between phylogenetic relationships uncovered in this study and previous taxonomic classifications for *Astyanax *and *Bramocharax *from Mesoamerica [35,37,52] is not surprising. In addition, our results are not in agreement with the idea that *Astyanax *(including samples from Mexico and Upper Central America) is a single species (i.e., *A. fasciatus*) as has previously proposed [37].

Although not a main goal of this study, we propose a provisional taxonomic nomenclature for *Astyanax *populations from Mesoamerica (see Additional file 3). The nomenclature proposed is based on well-defined monophyletic groups, high genetic divergences with *Cytb *(>2% *K81uf*), and in agreement with geographical distributions. In ascribing species names we gave priority to previous species descriptions and diagnostic morphological traits. Where monophyletic lineages could not be assigned to a valid species name, they were assigned to their own monophyletic group as *Astyanax sp*.

### Genetic and Time Divergences

The penalized likelihood analyses performed in r8s for *Cytb *sequences was calibrated using the following events: 1) the Merida-Perija uplift about 8–12 Mya, 2) the presence of fossils of *Colossoma macropomum *in the Magdalena basin (from at least 15 Mya), and 3) the formation of the TMVB about 3–6 Mya (Figure 6) [53]. The analysis gave an average divergence rate of 0.8% per million years with our in-group and the *K81uf *model of evolution (Figure 6). While this is similar to divergence rates reported for other fishes [Cichlidae (0.7%), [22], Cobitidae (0.68%) [54] (Table 3) and slightly lower than in cyprinid fishes (1.05%) [38], it is much lower than previous molecular clock rates (using fragments from the same gene) proposed for *Astyanax *(1.5% *K2P *divergences) [37]. This difference in estimated divergence rate is partially the cause of discrepancies between our study and previous historical biogeographical interpretations for *Astyanax *[37].

**Table 3:** Genetic distances in percentage among major groups (below diagonal, uncorrected *p *sequence divergences; above diagonal, ML distances *K81uf*)

	**Between**						**Within**	
	
	1	2	3	4	5	6	Uncorrected	GTR
Group I	-	5.74	5.68	4.77	6.44	6.37	2.84	3.25
Group II	4.65	-	5.4	4.73	6.1	5.89	2.51	2.86
Group III	4.64	4.4	-	4.58	6.79	5.58	0.79	0.82
Group IV	4.05	4.03	3.91	-	5.45	5.27	0	0
Group V	5.19	4.91	5.41	4.51	-	7.05	0.23	0.23
GroupVI	5.08	4.77	4.57	4.41	5.6	-	0	0

### Biogeographical implications

We found a pattern of north-south phylogeographical structuring. The major phylogenetic groups were mostly non-overlapping, with the exception of Groups I and II, which overlap in the upper part of the Polochic basin of Guatemala, and Groups II and III, which overlap in the Ciruelas basin of Costa Rica. This north-south pattern is similar to that reported for other freshwater fishes [12,13,22]. We explain the observation of sympatric lineages of *Astyanax *in terms of niche overlap and lineage turnover, similar to that proposed in biogeographical models for other characins in Mesoamerica [15].

The lack of phylogenetic structuring in *Astyanax *in Middle Central America (even with the subset of data 3.8 Kbp) can be explained by a more recent colonization and rapid radiation about 6.9 Mya (Table 4); this pattern has been previously observed in other freshwater fishes of the region [12,13,15,22,55].

**Table 4:** Summary of the timings of the main geological events in Mesoamerica based on freshwater fauna.

*Genera*	*Gene*	*Calibration %/Mya*	*Event*	*Event Time*
*Roeboides*, *Hypopomus *and Pimelodella* [8]	ATP6&8	1.3% K2P		4–7 Mya Mio-Pliocene
Astyanax* [37]	*Cytb*	1.5% *K2P*		3 Mya Pliocene
Rhamdia* [12]	*Cytb *+	1.3% *K2P*		2.5–2.9 Mya guatemalensis clade
	*ATP6&8*	1.5% (*Cytb*) *HKY85*		6.5–5.6 Mya laticauda clade
				Mio-Pliocene
Synbranchus and Ophisternon** [13]	*Cytb *+	1.3% (*ATP6&8*) *K2P*		12.7 – 23 Mya Miocene
	*ATP6&8*	1.5% (*Cytb*) *TrN+G*	*Colonization of the Mesoamerica from South America*	
Cichlidae** [22]	*Cytb*	0.7% uncorrected		10 Mya Miocene
Rivulus**[86], recalculated	*Cytb*	1% uncorrected		18–20 Mya Miocene
Brycon, Bryconamericus, Eretmobrycon and Cyphocharax * [15]	*ATP6&8*	3.6% K*s*		< 3.1 Mya Plio-Pleistocene
Poeciliidae [57]	MtDNA and nuclear genes	Mateos (2002) calibration		Before to Panama closure Cretaceous-Miocene
Poeciliopsis** [11]	*Cytb*	1–2% *K2P*	TMVB	8–16 Mya to 2.8–6.4 Mya Mio-Pliocene

*Primary freshwater fishes, ** Secondary freshwater fishes.

### Dispersal hypothesis on the origin of genus *Astyanax *in Mesoamerica

We accept the widely held hypothesis of a South American origin for *Astyanax *and other Central American characids [15,30,33]. This is supported by the observation that Lower Central America lineages were most closely related to South American samples from Brazil and Argentina. Considering the widely used *Cytb *calibration rate for fish (1.09%/my *HKY *distances) [22,38,54] and our mean rate of 0.8%/my *K81uf *distances from R8s (Figure 6 and Table 4), levels of divergence for populations of South and Central America imply a period of Mesoamerican colonization/expansion of *Astyanax *from South America about 7.8–8.1 Mya, before the final uplift of the Isthmus of Panama ~3.3 Mya [7,20,56]. The inclusion of more *Astyanax *samples from both sides of the Sierra de Perija and Merida Andes in further studies could improve this dating scenario.

The colonization of Central America prior to Late Cenozoic closure of the Panama Strait is incongruent with the geological data, and with other studies of characid genera (*Brycon*, *Bryconamericus*, *Eretmobrycon *and *Cyphocharax*) [15], including a previous study of *Astyanax *[37], all of which propose that closure of the strait ~3.3 Mya provided the first opportunity for colonization of Central America from South America.

An earlier colonization of Mesoamerica has been proposed for other freshwater fishes (Table 3) [[8,12], [13], [22]]. For example, ancient colonization events have been proposed for the family Poeciliidae (Cretaceous – Rosen model [23]) [57] and the Cichlidae, Rivulidae and Synbranchidae families (Miocene – GAARlandia model as proposed by Iturralde-Vinent and MacPhee [7]) [8,12,13,22]. However in contrast with *Astyanax*, these families also occur in the Caribbean islands, and as secondary fishes could have crossed through a shallow passage between South America and Central America during the Miocene [13,20,22].

Divergence times similar to those found in this study have been reported in molecular studies of primary freshwater fauna [8,12]. For example, the Bermingham and Martin model [8] proposes a colonization of Mesoamerica between 4 and 7 Mya, prior to the final closure of the Panama Isthmus (~3.3 Mya), based on comparative phylogeography of three genera [*Roeboides *(Characidae), *Pimelodella *(Pimelodidae) and *Hypopomus *(Hypopomidae)] Moreover, *Rhamdia *seems to have colonized Mesoamerica in two different waves, one prior to the closure of the Panama Strait (6.5 to 5.6 Mya, *R. laticauda *group) [12]. These estimates are in agreement with our study, and coincident with other primary and secondary freshwater fish fauna [12,13,22], as well as divergence times for invertebrates (9 Mya in pseudoscorpions) [26] and benthic foraminifera fossils (8 Mya) [58].

We found evidence of a unique biogeographical pattern involving multiple waves of expansion of Group I (Clades II – V) *Astyanax *in the upper part of the Polochic-Motagua fault (Mexico and Chiapas region). These clades have a restricted distribution overlapping that of Clade I, and in general occupy relatively stable ecological environments (springs or lakes), which can be less affected by climate change. Niche overlap and lineage turnover could explain this pattern, except in stable habitats where two lineages are found in sympatry (lineages Ia of Clade I and the lineage of Clade II in the Huasteca region, and in the Mezquital and Nazas-Aguanaval basin).

### Main vicariant events in *Astyanax *populations from Mesoamerica

The vicariance events involving *Astyanax *in Mesoamerica occurred during the Plio-Miocene (4–8 Mya), occurring earlier in Lower Central America (Panama and mainly Costa Rica) than in Central and Upper Mesoamerica.

A pattern of restricted geographic ranges in Lower Central America (Groups IV-VI) supports pronounced geographical fragmentation as a consequence of tectonics movements [7,20], which eventually resulted in closure of the Panama Strait ~3.3 Mya. Bermingham and Martin [8] have implicated multiple range fragmentation during the Miocene in patterns of diversity in other taxa of the primary freshwater fauna. With our data, five main vicariant events were identified for Lower Central America (Figure 6). These are related to changes in eustatic sea level (5–8 Mya) [25] and to the formation of inter-oceanic biogeographical barriers [58] during the Middle-Late Miocene (8 Mya).

In Upper Central America, the main volcanic activity was produced by the Trans-Mexican Volcanic Belt (TMVB). This region was affected by periods of intense geological activity between 3 and 12 Mya, with some volcanic activity still occurring today [53,59,60]. The geographic structuring evident in Clade I of *Astyanax *indicates that the TMVB formed an effective geographic barrier during its development during the late Miocene 4 – 6 Mya (Figure 6). This date is in agreement with the geology of the region and previous studies of several groups of vertebrates [1,5,10,11,61,62].

### Other biogeographical patterns

Other biogeographical patterns were obtained in Lower and Middle Central America. These cannot be explained by geological barriers, but are in accordance with the main biogeographical regions proposed for other freshwater fishes [15,22,63].

While our results contrast somewhat with the Mesoamerican faunal regions recognized by Bussing [14], they do support his Isthmian Region, but with a fragmented pattern similar to that reported for other primary freshwater fishes [8,12,15]. Furthermore, we found that some Belize and Guatemala *Astyanax *clades (Clades VII and VIII in Figure 5) were joined to the Central America group (group II), a pattern shared with other Mesoamerican Cichlids [22]. Our Chiapas-Nicaraguan lineages (Lineage Id in Figure 4) did not reach the Pacific cost of Costa Rica, but instead have their southern limits in El Salvador. The distribution of lineages from San Juan Region differs from Bussing's proposal [14], occurring from the Barbilla basin (Costa Rica) to Belize (Atlantic slope) and on the Pacific slope from the Rio Grande Basin to the Ciruelas basin in the Nicoya Gulf (Costa Rica). This pattern has been previously reported by other authors [22,63].

Finally, we dated the separation of Groups I and II to about 6 and 7.8 Mya (Table 4). These coincide geographically with Polochic-Motagua Fault [6], reported as a transition region for other freshwater fish groups [22,13]. In addition, we observed the presence of two well differentiated Lineages of *Astyanax *(Clade IV in Group I and Clade V in Group II) in sympatry in the Polochic basin, a finding that has not been reported for other characids [15]. We explain this pattern in terms of river capture whereby the Cahabon tributary was diverted to the Polochic river as a consequence of tectonic activity (Sierra de Chiapas [64]), while separating the Cahabon river from the Grijalva-Usumacinta (Clade IV in Group I). This has been proposed for *Rhamdia *[12].

## Methods

### Tissue collection and DNA extractions

A total of 208 specimens of the *Astyanax *and *Bramocharax *from 141 localities from Panama to the Mexican-USA border (Additional file 3; Figure 3) were analyzed, corresponding to 10 species of *Astyanax*: *A. aeneus*, *A. altior*, *A. armandoi*, *A. fasciatus*, *A. jordani*, *A. mexicanus*, *A. nasutus*, *A. nicaraguensis*, *A. orthodus *and *A. petenensis*; and three species of *Bramocharax*: *B. caballeroi*, *B. dorioni *and *B. bransfordii*. Samples of *Roeboides bouchellei *(from El Salvador), *Astyanax bimaculatus *(from Argentina) and *Astyanax fasciatus *(GenBank sequence from Brazil) were used as outgroups. We collected individuals from the type localities of most of the species (10 nominal species) described in Mesoamerica and considered valid by Lima *et al *[35]. Specimens were sampled by electro-fishing and netting, individually tagged, and preserved in DMSO/EDTA buffer [65] or 95% ethanol. DNA voucher specimens and their associated lots were subsequently preserved in 10% buffered formalin and deposited in the Museo Nacional de Ciencias Naturales of Madrid, Spain (MNCN), and the Universidad Michoacana de San Nicolas de Hidalgo, Michoacan, Mexico (UMSNH, see Additional file 3).

### DNA extraction and Sequencing

Genomic DNA was isolated by standard proteinase K and phenol/chloroform extraction methods [66] and stored at 4°C. The entire cytochrome *b *(*Cytb*) gene (1140 bp) and fragments of 16 S rRNA (552 bp) and cytochrome oxidase I (*COI*) (655 bp) genes were amplified. We also amplified exon 3 of the nuclear Recombinant Activating Gene 1 (*RAG1*) (1512 bp). Polymerase chain reactions (PCRs) were performed in 25-μL reactions containing 0.4 μM of each primer, 0.2 μM of each dNTP, 2 mM MgCl_2_, and 1.5 units of *Taq *DNA polymerase (Biotools). PCRs were conducted under the following conditions: 94°C (2 min), 35 cycles of 94°C (45 s), region specific Tm°C (1 min), 72°C (90 s), and 72°C (5 min), for most amplifications (see Table 1), with the exception of the *RAG1 *gene for which we followed the PCR conditions described in [67]. PCR products were run on 1.0% agarose gels to confirm amplification and purified with the EXOSAP-IT PCR Product Clean – Up (Usb) kit or by ethanol precipitation. Both strands were sequenced (see Table 5 for primers) and run on an ABI 3700 DNA automated sequencer (SECUGEN sequencing service).

**Table 5:** Estimated dates derived from the r8s molecular dating analyses, along with standard deviations of the node ages derived from Penalized Likelihood bootstrap analyses.

		*Estimated ages*		
		
*Node*	*Calibration point*	Strict Molecular Clock		Relaxed Molecular Clock NPRS with r8s Mya
		0.8%/Mya	1.05%/Mya	95% of confidence
1	Merida-Perija Mountains **8–12 Mya**	24.6 ML (18 – 23)	32.3 ML (23.7–42.8)	15
	*Colossoma macropomum ***15 Mya**	11 *p *(9.8 – 12.4)	14.6 *p *(12.8–16.3)	
2	Mesoamerica Colonization	7.7 ML (6.36–9.32)	10.05 ML (8.35–12.23)	7.9 SD 0.165 (7.8–8.1)
		5.5 *p *(4.9–6.4)	7.3 *p *(6.4–8.5)	
3	Motagua-Polochic fault	4.6 ML (2.9–7.9)	6 ML (3.8–10)	7.1* SD 0.34 (6.0–7.8)
		3.7 *p *(2.5–5.7)	4.9 *p *(3.3–7.5)	
4	Radiation in North Mesoamerica Lineages	3.8 ML (2–6.2)	4.83 ML (2.7–8.2)	6.7* SD 0.37 (5.7–7.5)
		3.1 *p *(1.9–4.8)	4.1 *p *(2.5–6.3)	
5	Central America Radiation	3.7 ML (2–6.2)	5 ML(0.2–7.42)	6.9* SD 0.39 (5.9–7.7)
		3.1 *p *(1.9–4.8)	4.2 *p *(0.2–5.5)	
6	TMVB **3–6 Mya**	3.6 ML (2.3–5.4)	4.7 ML (3.1–7)	5.25* SD 0.37 (4.4–6.2)
		3.07 *p *(2.2–4.3)	4.03 *p *(2.8–5.7)	

The fixed ages are shown in Bold. *P *= Uncorrected distances ML = *K81uf *distances. Minimum and maximum values are given in parenthesis. Asterisks identify values with a normal distribution p ≤ 0.05.

### Data analysis

Chromatograms and alignments were visually checked and verified. Saturation for transition and transversion substitutions was checked by plotting the absolute number of changes at each codon position against patristic distances for coding genes only.

Phylogenetic reconstruction was performed for Bayesian Inference (BI) using MrBayes version 3.1.2 [68]. We used Modeltest 3.07 [69] to find the best-fit model of evolution for each gene fragment using the Bayesian Information Criterion (BIC) [69] (see Additional file 4). BI was performed on two data sets as follows: (1) *Cytb *gene only with separate best-fit models for each codon position and (2) using the three mtDNA and *RAG1 *(separate best-fit models for each codon position were used) genes with a separate best-fit model of evolution for each gene fragment (partition). Analyses of best-fit models of evolution and BI were performed for a subset of the data.

Bayesian analyses were performed using two independent runs of four Metropolis-coupled chains of 10 million generations each to estimate the posterior probability distribution. The first 10,000 trees were discarded as burn-in. The program Tracer v1.4 [70] was used to assess run convergence and determine burn-in.

Sequence data were also analysed using maximum parsimony (MP) as implemented in PAUP* 4.0 b10 [71], NONA version 2.0 [72] and WINCLADA version 1.00.08 [73]. MP analyses in PAUP* and NONA/WINCLADA were done under the same heuristic search strategy. Statistical support for recovered clades was assessed using bootstrap (1000 pseudo-replications). We applied different weights for transversions and transitions according to the empiric criterion obtained in PAUP* 4.0 b10 [71]. The two datasets run for BI were also run for MP.

Analysis 1 (for which most species and populations of the *Astyanax *and *Bramocharax *genera from Mesoamerica were represented) was used to infer phylogenetic relationships among populations. Analysis 2 (for which only a subset of the species/populations were available) was used to infer relationships among the main lineages identified in analysis 1.

### Molecular clock and divergence time

Since we have a more complete data matrix, and in order to make our data comparable with previous studies in the region, we calibrated the molecular clock based on *Cytb *data alone rather than the combined data matrix (which included two more mitochondrial genes and one nuclear DNA gene).

Rate heterogeneity within the dataset was assessed using the likelihood-ratio test (LRT) [74,75]. LRTs were determined by comparing the log likelihood of the optimal topology recovered by maximum likelihood analysis (using appropriate models identified by Modeltest), while enforcing the molecular clock to the log likelihood of the optimal topology recovered by one that did not. The likelihood ratio statistic is twice the difference between the two log likelihoods. This statistic is compared to a χ^2 ^distribution with degrees of freedom equal to the number of terminals minus two following [76].

Because the molecular clock hypothesis was rejected, we conducted a non-parametric rate smoothing approach (NPRS) of divergence time estimation with the r8s package [75] to estimate divergence between the taxa. NPRS relaxes the molecular clock assumption by applying a least squares smoothing of estimates of substitutions rates.

Standard errors of divergence dates were estimated using the boot strapping procedure outlined in, and implemented by, Perl scripts in the r8s bootkit provided by Torsten Eriksson [77]. The first 100 bootstrapped datasets were created from the original *Cytb *dataset with the program Mesquite v. 2.01 [78]. Branch lengths were then re-estimated for each bootstrapped dataset in PAUP* using the original ML parameters. The resulting trees, with branch lengths, were then imported into r8s. The TN (Truncated Newton) algorithm was implemented.

We constrained the molecular clock considering two main points in the tree topology (see figure 6). First, our ingroup (node 1, Figure 6) was calibrated with the isolation of the Maracaibo basin as a result of the rise of Sierra de Perija and Merida Andes 8–12 Mya: [7,79,80]. Additionally for this node, we also used the oldest fossil record for a Characid (*Colossoma macropomum*, 15 Mya) in the Magdalena river system [81]. This allowed us to determine the minimum and maximum values for this node (8 and 15 Mya, respectively). We also constrained the molecular clock using the final closure of the Trans-Mexican Volcanic Belt (TMVB) 3–6 Mya (node 6 in the Figure 6) [53,60].

## Authors' contributions

CPOG collected material, compiled data, performed analyses and wrote the manuscript. ID designed the research project, collected material and wrote the manuscript. ODD collected material. The final draft was read and approved by all the authors.

## Supplementary Material

Additional File 1 **Sampling sites in Mesoamerica.** Sampling points, valid species *sensu *Lima *et al*. [35] and taxonomical proposals. The water body code is: R = River, C = Cenote (sink holes) A = Lagoon, L = Lake and S = spring. Click here for file

Additional File 2 **Parameters of ML analyses estimated for each gene with Modeltest **[69]. Maximum Likelihood parameters calculated by Modeltest [69], using the Bayesian Information Criteria (BIC): I = Proportion of invariable sites, Γ = Gamma, Ti/Tv= Transition/Transversion ratio. Click here for file

Additional File 3 **Genetic divergences between Lineages.** Genetic distances in percentage among Lineages (below diagonal, uncorrected p sequence divergences; above diagonal, ML distances *K81uf*) Click here for file

Additional File 4 **Groups and Lineages Scheme.** Main Groups Scheme. The zigzag lines represent vicariant events. MEX = Mexico, BEL = Belize, GUA = Guatemala, S = Sabinos, A = Aguanaval, M = Mezquital, R = Rascon, T = Tamasopo, POL = Polochic, GRI = Grijalva, USU = Usumacinta, MAQ = Maquinas, MON = Montebello and MOT = Motagua. Click here for file
