# Supplementary figures and images for: Evolutionary history of the fish genus Astyanax Baird & Girard (1854) (Actinopterygii, Characidae) in Mesoamerica reveals multiple morphological homoplasies
Source: BMC Evol Biol. 2008 Dec 22;8:340. doi: 10.1186/1471-2148-8-340 (PMC2657800; doi:10.1186/1471-2148-8-340)

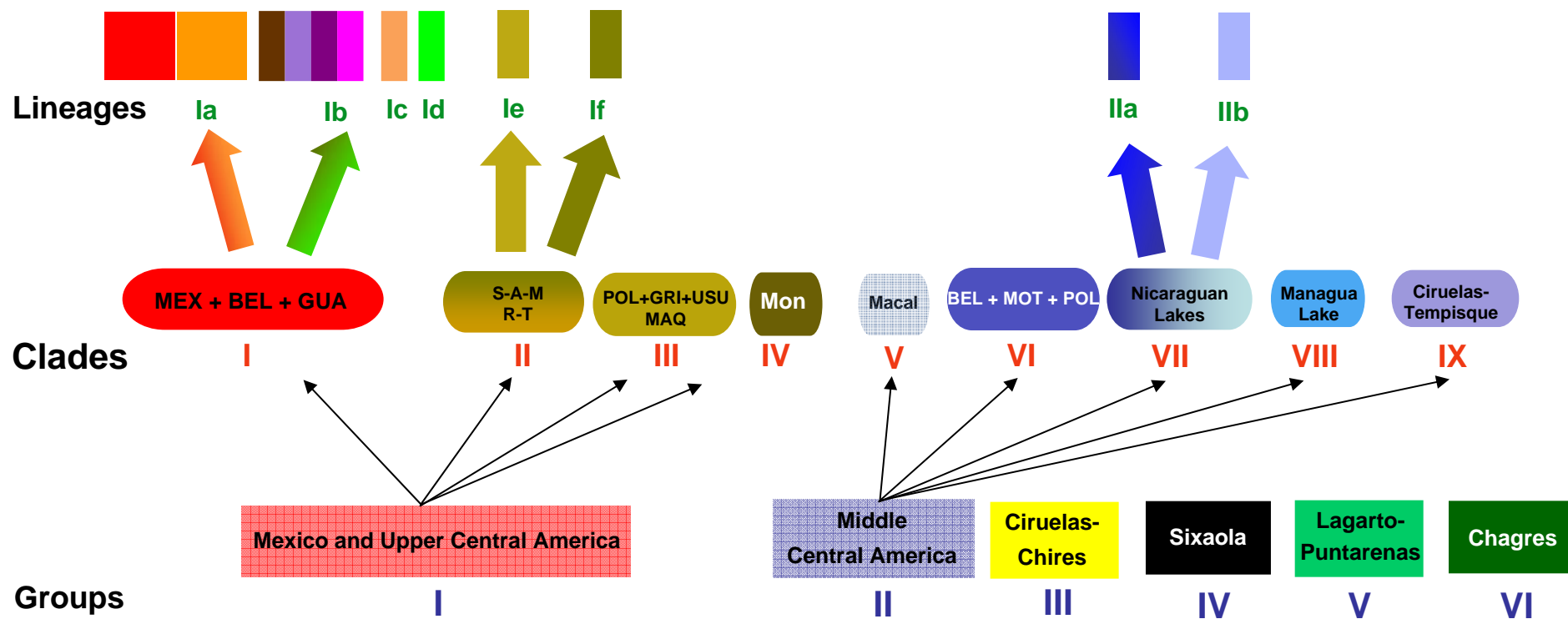

North America and Central America *Astyanax*

Supplement: Additional File 4 — Groups and Lineages Scheme. Main Groups Scheme. The zigzag lines represent vicariant events. MEX = Mexico, BEL = Belize, GUA = Guatemala, S = Sabinos, A = Aguanaval, M = Mezquital, R = Rascon, T = Tamasopo, POL = Polochic, GRI = Grijalva, USU = Usumacinta, MAQ = Maquinas, MON = Montebello and MOT = Motagua. [file 1471-2148-8-340-S4.pdf]
